# Pseudohyperkalemia in a Patient With Chronic Lymphocytic Leukemia

**DOI:** 10.7759/cureus.23512

**Published:** 2022-03-26

**Authors:** Rahul Gujarathi, Venu Chippa, Narsimha Candula, Meet Kadakia

**Affiliations:** 1 Hospital Medicine, University of Florida Health, Jacksonville, USA; 2 Internal Medicine, St. Vincent Medical Center, Evansville, USA; 3 Hematology and Medical Oncology, University of Florida College of Medicine, Jacksonville, USA

**Keywords:** reverse pseudohyperkalemia, refractory hyperkalemia, persistent hyperkalemia, chronic lymphocytic leukemia (cll), hyperleukocytosis, spurious hyperkalemia, pseudohyperkalemia

## Abstract

Hyperkalemia is a common electrolyte disorder with potentially life-threatening consequences, including cardiac dysrhythmias. Pseudohyperkalemia must always be ruled out before implementing treatment for true hyperkalemia. Here, we present a case of a 63-year-old male with chronic lymphocytic leukemia (CLL) with a white blood cell count greater than 200 thousand/mm^3^ and persistently high serum potassium concentration as high as 8.4 mmol/L. A venous blood gas analysis was performed, which confirmed the patient's plasma potassium levels were within the normal range (3.7-4.4 mmol/L). In patients with CLL, due to the increased fragility of their white blood cells, mechanical stress such as centrifugation can lead to cell lysis resulting in pseudohyperkalemia. Our emphasis with clinicians is to familiarize themselves with these spurious laboratory values and prevent unnecessary invasive testing and treatment.

## Introduction

Hyperkalemia is common in clinical medicine, and its treatment varies with underlying cause and comorbidities. True hyperkalemia can result from renal dysfunction, drug effects, endocrinopathies, and diet changes. Pseudohyperkalemia (spurious hyperkalemia) refers to the false elevation in the measured serum potassium concentration caused by the movement of potassium out of the cells during or after the blood sample has been collected [1]. Pseudohyperkalemia is confirmed by determining plasma potassium in vacuum tubes with lithium heparin (e.g. blood gas syringe) after centrifugation and by determining whole blood potassium in an electrolyte-balanced lithium heparin syringe [2]. Spurious hyperkalemia is usually related to the blood drawing technique but can also occur in patients with markedly elevated white blood cell or platelet counts. The normal potassium level in serum is 0.4 mmol/L higher than that of plasma due to potassium release during clot formation. Pseudohyperkalemia should only be considered when the serum potassium concentration exceeds plasma by at least 0.4 mmol/L [3]. Given the diverse etiology of hyperkalemia, it is essential to recognize and differentiate between true hyperkalemia and pseudohyperkalemia before performing further investigations and administering treatment.

## Case presentation

A 63-year-old male with a medical history of hypertension, chronic lymphocytic leukemia (CLL), chronic hepatitis C, and human immunodeficiency virus (HIV), on highly active antiretroviral therapy (HAART), presented to the hospital with a complaint of progressively worsening dyspnea, aggravated with exertion and affecting his daily activities. The patient's medication list included carvedilol, HAART (bictegravir/emtricitabine/tenofovir alafenamide), and lisinopril. The patient denied any other complaints but gave a history of sick contacts. The patient's initial vital signs included a temperature of 97.7˚F, a pulse rate of 88 beats per minute, a blood pressure of 106/97 mmHg, a respiratory rate of 20 breaths per minute, and oxygen saturation of 89% on room air. His physical exam was significant for bilateral pulmonary rhonchi on auscultation. Subsequently, he was admitted for acute respiratory failure with hypoxia due to coronavirus disease 2019 (COVID-19). He was treated with dexamethasone (6 mg oral tablet once daily for 10 days) and provided oxygen supplementation resulting in clinical improvement of his respiratory status.

**Figure 1:**
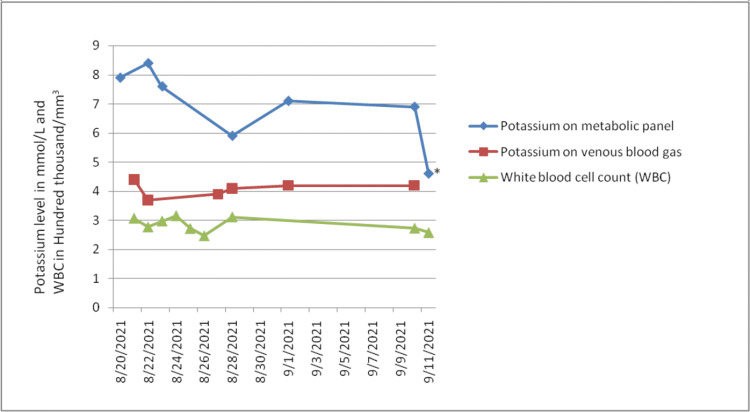
Graph depicting potassium levels and white blood cell count of the patient. * represents the whole blood potassium level drawn in an electrolyte-balanced lithium heparinized tube.

**Figure 2:**
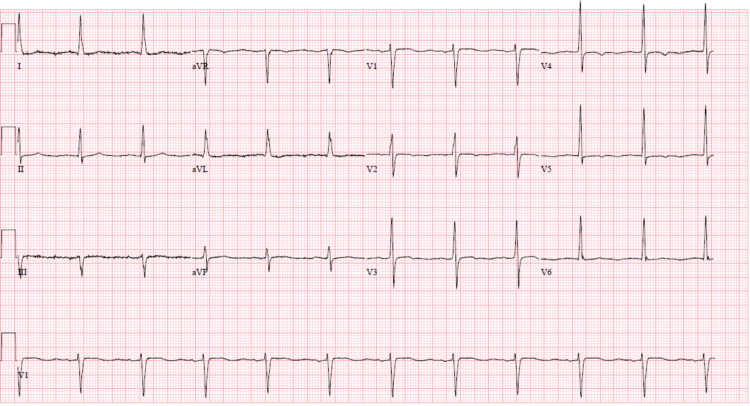
Electrocardiogram showing normal sinus rhythm.

Laboratory tests at presentation showed white blood cells (WBCs) of 307.34 thousand/mm^3^ (absolute lymphocyte count of 293.01 thousand/mm^3^), consistent with his CLL, and a critically high serum potassium level of 7.9 mmol/L. The patient's potassium levels and WBC count remained persistently high, as shown in Figure 1. His renal function and other electrolytes like phosphorus, calcium, magnesium, bicarbonate, and uric acid were unremarkable. His lactate dehydrogenase (LDH) was 255 IU/L (reference range: 126-266 IU/L) and thyroid-stimulating hormone (TSH) was 1.36 mIU/L (reference range: 0.27-4.2 mIU/L). His electrocardiogram revealed normal sinus rhythm without any T-wave changes, as shown in Figure 2. He had chronic hyperleukocytosis due to CLL and showed no signs of leukostasis during the admission. The patient's lisinopril was discontinued at admission. Due to high serum potassium levels, he received multiple doses of sodium polystyrene sulfonate and was placed on a renal diet with no significant improvement in his potassium levels. Nephrology was consulted for evaluation with a potential plan to get a tunneled central venous catheter to initiate hemodialysis for refractory hyperkalemia. Venous blood gas sample collected in a heparinized syringe confirmed the patient's serum potassium level of 4.4 mmol/L. This suggested that the patient's hyperkalemia was spurious due to his severe leukocytosis secondary to CLL.

## Discussion

Diagnosis of pseudohyperkalemia should be suspected when there is no apparent cause for the hyperkalemia in an asymptomatic patient, especially with no electrocardiographic manifestations of hyperkalemia. In our patient, causes of true hyperkalemia such as acute renal failure, tumor lysis syndrome, and acidosis were ruled out based on the normal renal function, phosphorus, LDH, uric acid, and bicarbonate levels. A retrospective observational study conducted at Shaare Zedek Medical Center (Jerusalem, Israel) showed a relatively high incidence of pseudohyperkalemia among CLL patients (accounted for 40% of all hyperkalemic episodes). It also reported that pseudohyperkalemia was characterized by significantly higher WBC counts and higher potassium and LDH levels compared to true hyperkalemia. Documentation of pseudohyperkalemia in medical charts was done only in a minority of cases [2].

Hyperleukocytosis with WBC count > 120 thousand/mm^3^ caused by CLL (as in our patient) can lead to falsely elevated potassium levels due to cell fragility and lysis. Centrifugation of a tube with a blood sample causes in vitro cell destruction resulting in potassium release as the cells are freely suspended in plasma [1]. Another phenomenon referred to as reverse pseudohyperkalemia observed in CLL patients is described as falsely higher potassium levels in plasma relative to serum. It is postulated to be due to the clotting process, which locks the leukocytes in the clot, thus curtailing the cell lysis [2]. Accurate assessment of the potassium concentration can be achieved by allowing clotting to separate serum from the cells before centrifugation. The use of point-of-care blood gases analysis has been previously reported to be an effective tool for detecting pseudohyperkalemia [3,4].

Venipuncture can cause mechanical trauma resulting in potassium release from red cells and a characteristic reddish tint of the serum due to the associated release of hemoglobin. Severe intravascular hemolysis can also present as red serum, similar to a hemolyzed specimen [5]. The measured serum potassium may denote the actual circulating value in patients with intravascular hemolysis. In patients with leukocytosis due to lymphoma or leukemia, spurious hyperkalemia has also been described after mechanical disruption of WBCs in the course of transporting the blood samples via pneumatic tube systems [6,7]. Repeated fist clenching during blood collection can raise the serum potassium level by more than 1-2 mmol/L in that forearm [8]. Venipuncture without trauma, a tourniquet, and repeated fist clenching will demonstrate the actual serum potassium concentration [9]. Prolonged length of storage and cooling of the serum sample can also increase serum potassium concentration [10,11]. In patients with familial (hereditary) forms of pseudohyperkalemia, potassium can exit the erythrocytes cells after the specimen is collected due to a rise in the passive potassium permeability of the red blood cells. Pseudohyperkalemia may be accompanied by abnormal red cell morphology in disorders like stomatocytosis.

## Conclusions

This case highlights that hyperleukocytosis in CLL patients can lead to pseudohyperkalemia due to increased cell fragility. Our goal is to increase awareness of this phenomenon among practicing physicians to prevent unnecessary testing and treatment with potassium lowering agents to avoid potentially lethal iatrogenic hypokalemia. True hyperkalemia in the absence of symptoms and overt kidney dysfunction must always be viewed with significant circumspection and scrutiny.
